# Supplementary material for: FGF18 alleviates hepatic ischemia-reperfusion injury via the USP16-mediated KEAP1/Nrf2 signaling pathway in male mice
Source: Nat Commun. 2023 Sep 30;14:6107. doi: 10.1038/s41467-023-41800-x (PMC10542385; doi:10.1038/s41467-023-41800-x)
Supplement: Supplementary file 1 — Supplementary Information [file 41467_2023_41800_MOESM1_ESM.pdf]

Supplementary information

FGF18 alleviates liver ischemia reperfusion injury via USP16 mediated KEAP1/Nrf2 signaling pathway

Tong *et al.*

Supplementary Figure 1

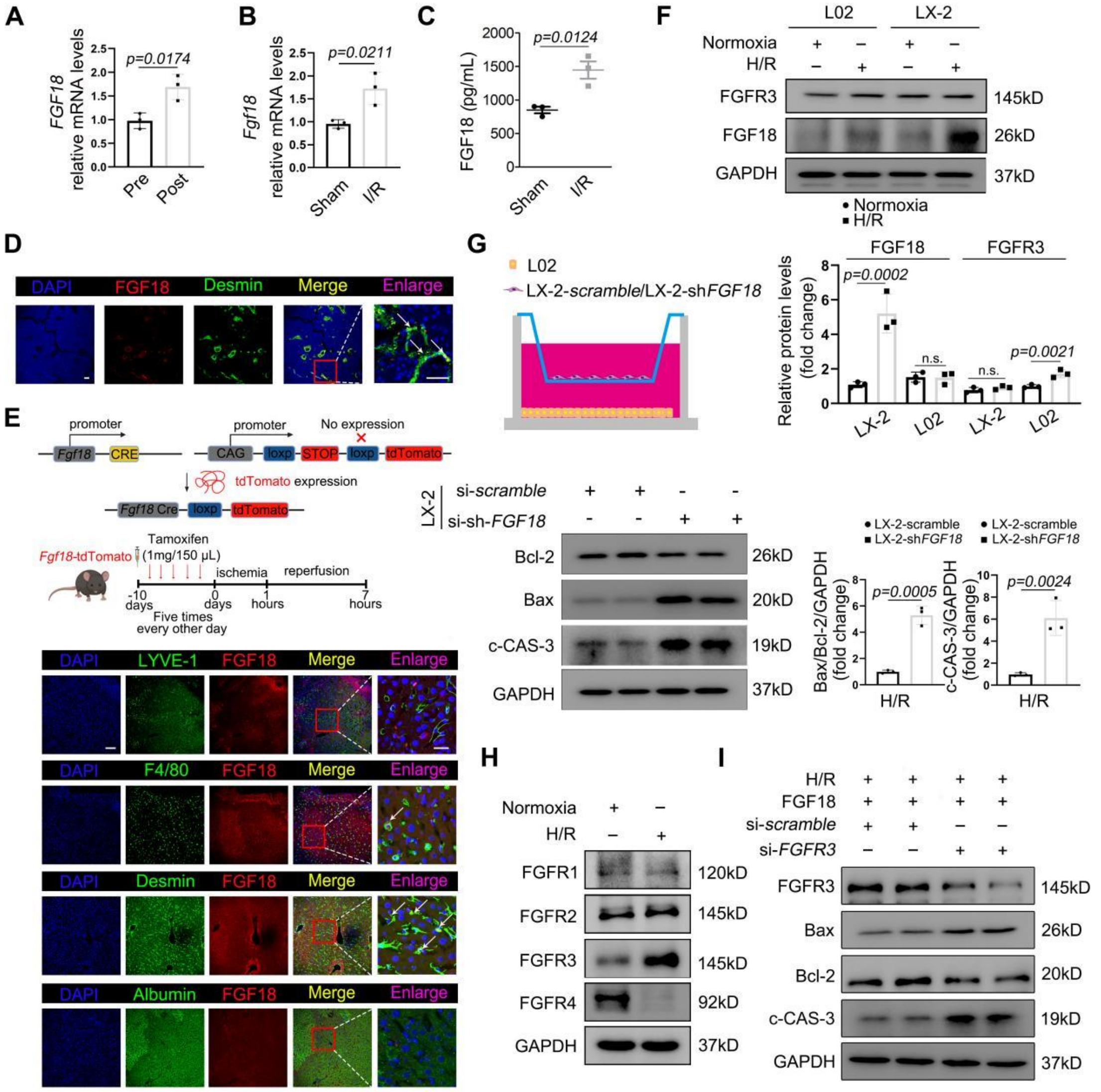

Supplementary Figure 1 (A-B) Relative mRNA expression of genes in the indicated group by RT-PCR (n=3). (C) The serum level of FGF18 in mice subjected to I/R (1 h/6 h) surgery or not (n=3). (D) Immunofluorescence for FGF18 (red) and desmin (green) (n=5). Scale bar=50 μm. The white arrows indicate the position of FGF18 together with the relative markers. (E) To induce *Fgf18-tdtomato* expression, mice were injected with tamoxifen (2 mg dissolved in 150 μL Corn Oil) every other day for a total of five times prior to the I/R (1 h/6 h) surgery. Immunofluorescence for

Tdtomato (red) and different cell markers (green) (n=5). Scale bar=50 μm. The white arrows indicate the position of FGF18 together with the relative markers. (Scheme is Created with BioRender.com) (F) L02 cells and LX-2 cells were subjected to H/R (4 h/6 h) treatment. Relative protein expression by western blotting (n=3). **(**G**)** Transwell assay was performed. Relative protein expression by western blotting (n=3). **(**H**)** Protein levels of FGFR1, FGFR2, FGFR3, and FGFR4 in L02 cells subjected to H/R (4 h/6 h) challenge or not (n=3). (I) Relative protein expression by western blotting (n=3). The statistical significance of differences were assessed by two-tailed student unpaired t-test for A-C, G. Other assays were assessed by one-way ANOVA, followed by Tukey's multiple comparison test. Data are presented as means ± SEM with individual values. All numbers (n) are biologically independent experiments. Source data are provided as a Source Data file.

Supplementary Figure 2

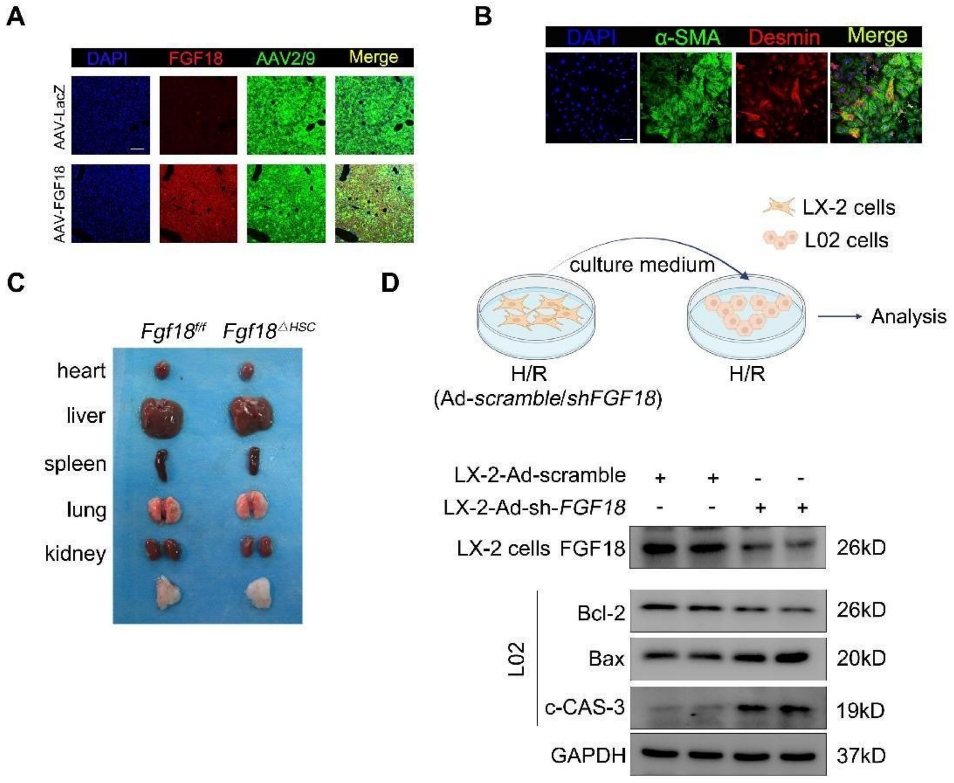

Supplementary Figure 2 (A) Immunofluorescence showed the transfection efficiency of AAV-FGF18 in liver mice sections (n=3). Scale bar: 100 μm. (B) Morphology of different organs from *Fgf18*$^{f/f}$ and *Fgf18*$^{\Delta HSC}$ mice. (C) HSCs were extracted from *Fgf18*$^{f/f}$ and *Fgf18*$^{\Delta HSC}$ mice subjected to I/R (1 h/6 h) surgery. Immunofluorescence for α-SMA (green), desmin (red), and DAPI (blue) (n=5). Scale bar: 100 μm. (D) schematic representation of the cell-based supernatant transfer assay (n=3). Relative protein expression by western blotting. (Scheme is Created with BioRender.com) Data are presented as means ± SEM with individual values. All numbers (n) are biologically independent experiments. Source data are provided as a Source Data file.

Supplementary Figure 3

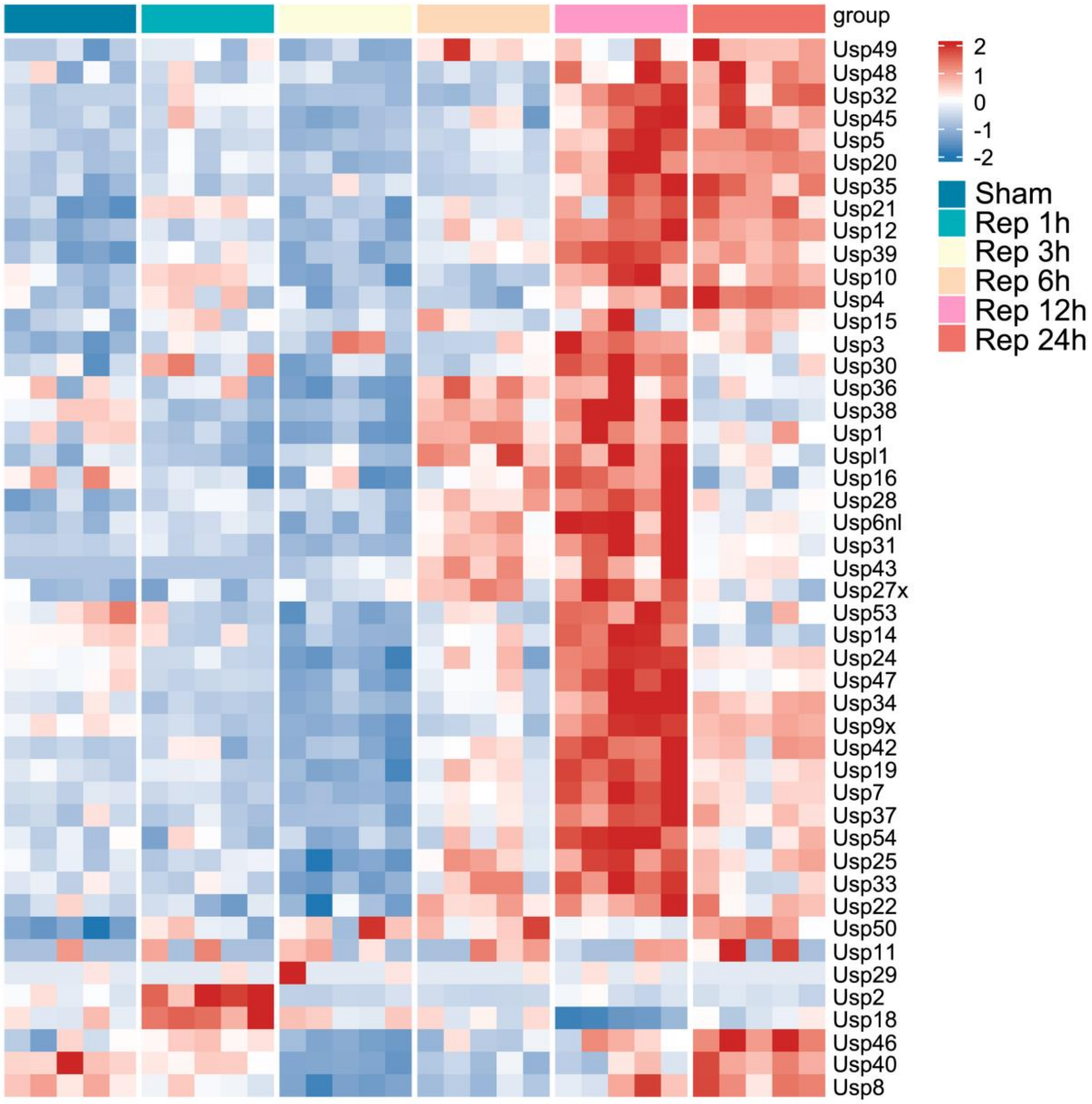

Supplementary Figure 3 (A) RNA-sequence was carried out between Sham and I/R with different reperfusion times (0, 1, 3, 6, 12, 24). The results of USP family changes were presented in the form of heat maps (n=5 per group).

Supplementary Figure 4

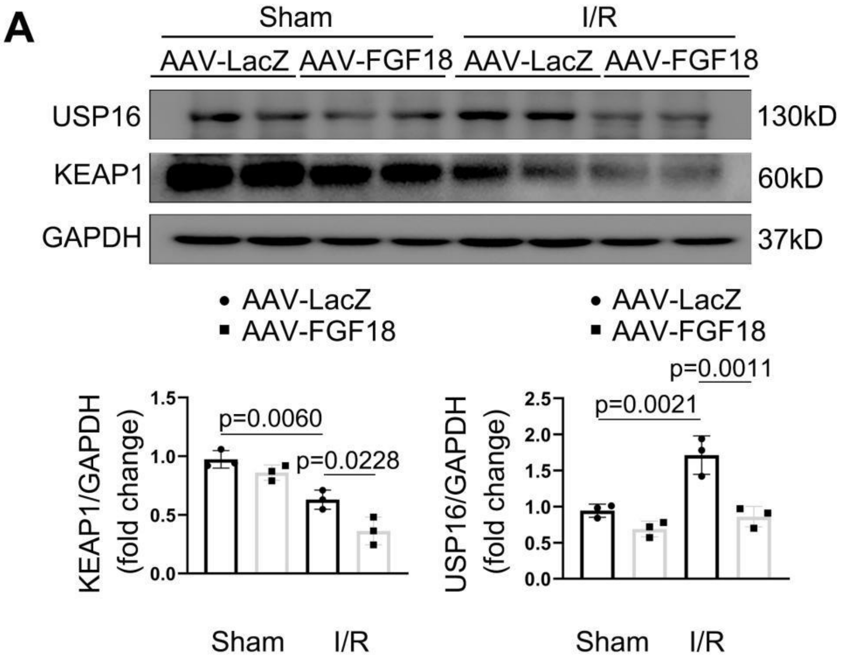

Supplementary Figure 4 (A) Protein levels of USP16 and KEAP1 in AAV-LacZ and AAV-FGF18 mice subjected to I/R (1 h/6 h) surgery or not (n=3). The statistical significance of differences were assessed by one-way ANOVA wherever applicable, followed by Tukey's multiple comparison test. Data are presented as means ± SEM with individual values. All numbers (n) are biologically independent experiments. Source data are provided as a Source Data file.

Supplementary Figure 5

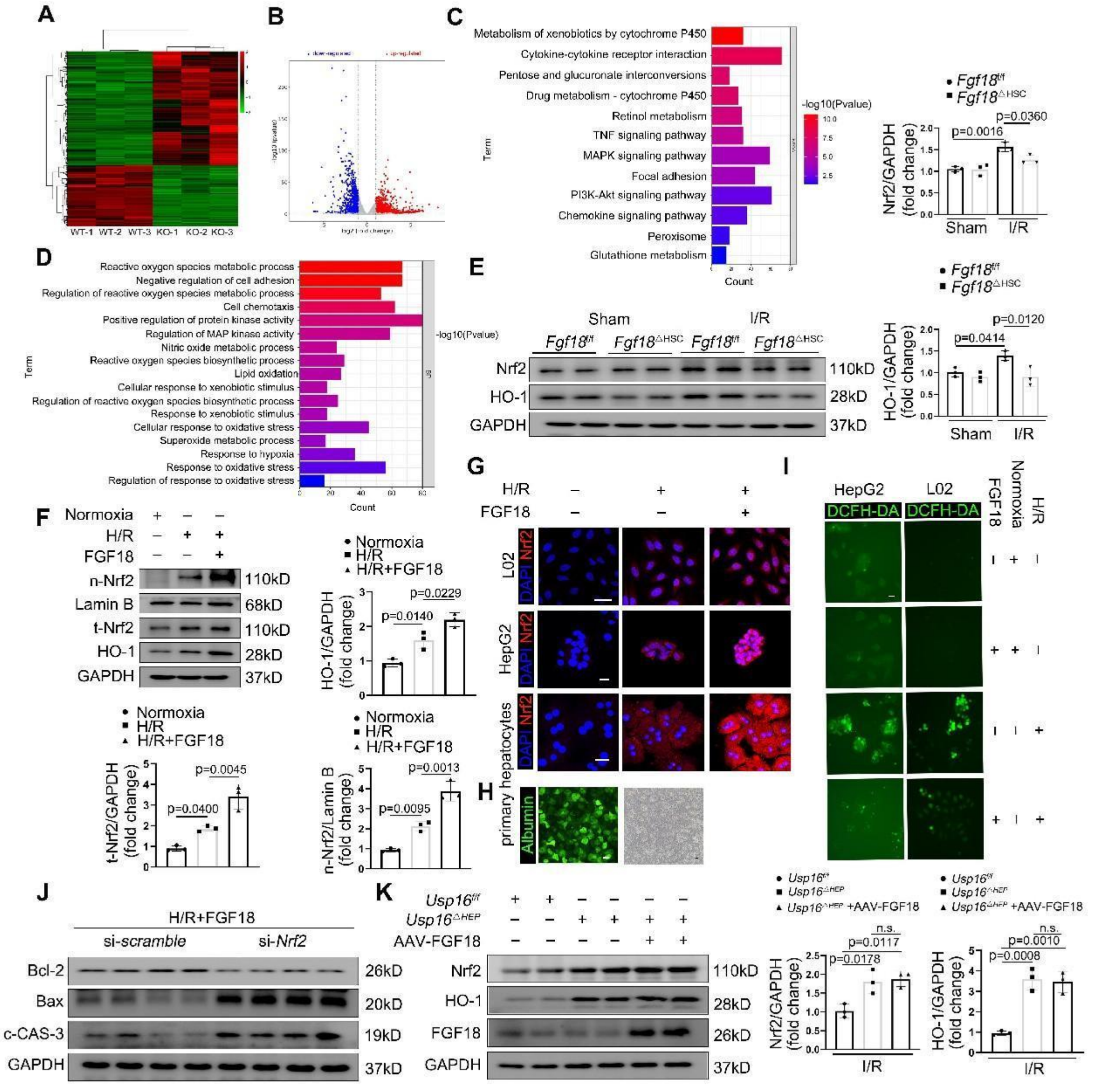

Supplementary Figure 5 FGF18 activates Nrf2 signaling pathway during hepatic IRI. (A) A heatmap representation of genes from *Fgf18*$^{f/f}$ mice and *Fgf18*$^{\triangle HSC}$ mice. (B) Volcano plots representation of significantly up-regulated (red) and down-regulated (blue) genes between *Fgf18*$^{f/f}$ mice and *Fgf18*$^{\triangle HSC}$ mice (n=3). (C-D) KEGG and BP. (E) Relative protein expression by western blotting (n=3). (F) Protein levels of (total) t-Nrf2, (nuclear) n-Nrf2, and HO-1 in FGF18 (200 ng/mL) treated L02 cells subjected

to H/R (4 h/6 h) challenge or not (n=3). (G) Nrf2 immunofluorescence staining in H/R (4 h/6 h) treated L02 cells, HepG2 cells, primary hepatocytes in the presence of FGF18 or not (n=5). Scale bar=50 μm. (H) The purity of primary hepatocytes was verified by the immunofluorescence of albumin and the bright field photoed by inverted microscope (n=5). (I) DCFH-DA staining (n=5). Scale bar=100 μm. (J) L02 cells were transfected with si-*Nrf2* or Scramble, and then subjected to H/R (4 h/6 h) challenge in the presence of FGF18 (n=3). (K) Relative protein expression by western blotting (n=3). The statistical significance of differences were assessed by one-way ANOVA wherever applicable, followed by Tukey's multiple comparison test. Data are presented as means ± SEM with individual values. All numbers (n) are biologically independent experiments. Source data are provided as a Source Data file. (n.s., not significant.)

Supplementary Figure 6

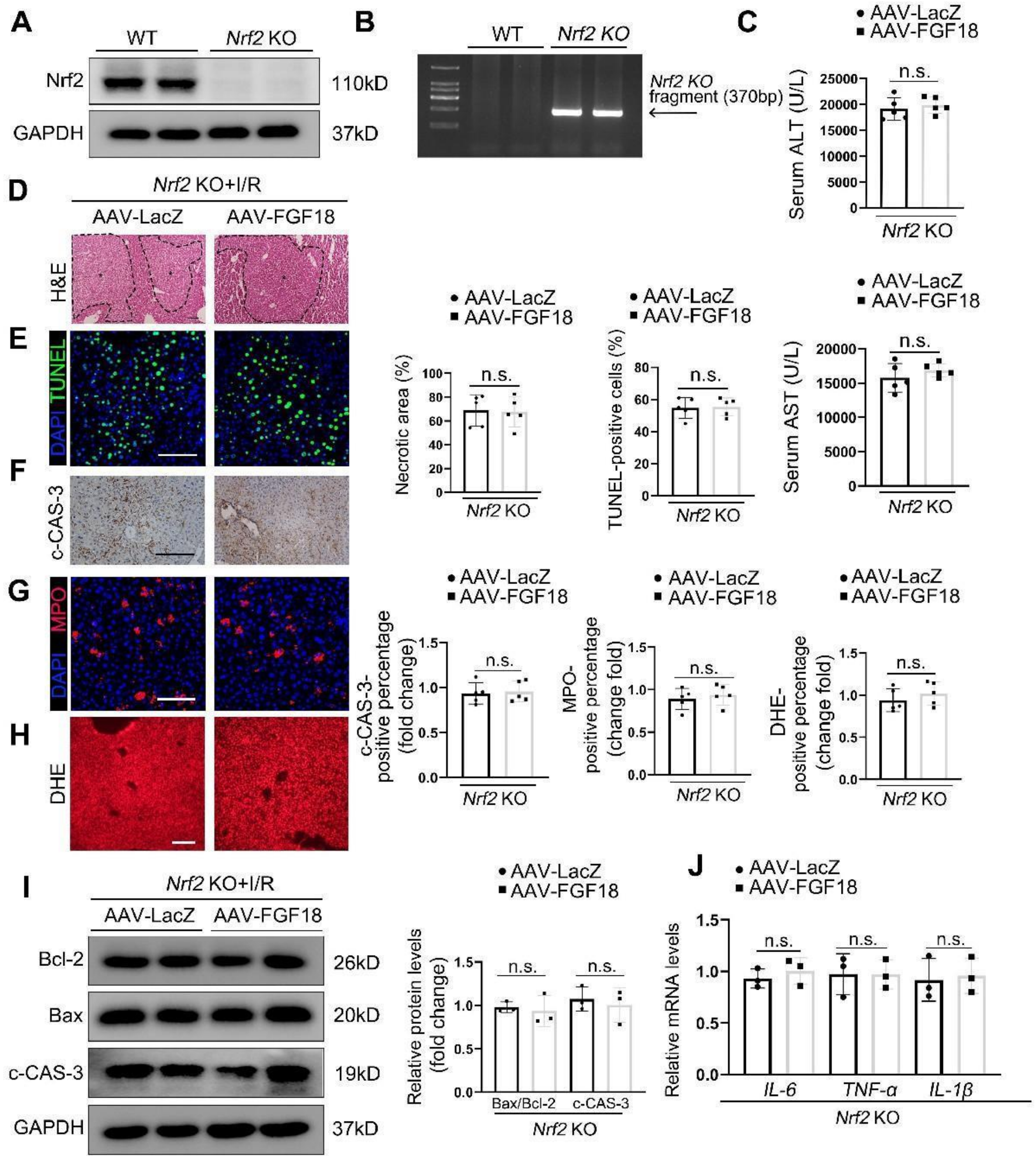

Supplementary Figure 6 FGF18 activates Nrf2 signaling pathway during hepatic IRI (A) Relative protein expression by western blotting. (B) The genotype of *Nrf2 KO* mice were confirmed by RT-PCR (n=3). (C) Serum ALT and AST level (n=5). (D) Representative H&E staining of liver sections from *Nrf2 KO* and *Nrf2 KO*+AAV-FGF18 mice subjected to I/R (1 h/6 h) surgery. H&E staining of liver

sections (n=5). Scale bar=100 μm. (E-H) TUNEL staining, c-CAS-3 immunohistochemistry, MPO, and DHE staining of liver sections (n=5). (I) Relative protein expression by western blotting (n=3). (J) Relative mRNA expression of genes by RT-PCR (n=5). The statistical significance of differences were assessed by two-tailed student unpaired t-test. Data are presented as means ± SEM with individual values. All numbers (n) are biologically independent experiments. Source data are provided as a Source Data file. (n.s., not significant.)

Supplementary Figure 7

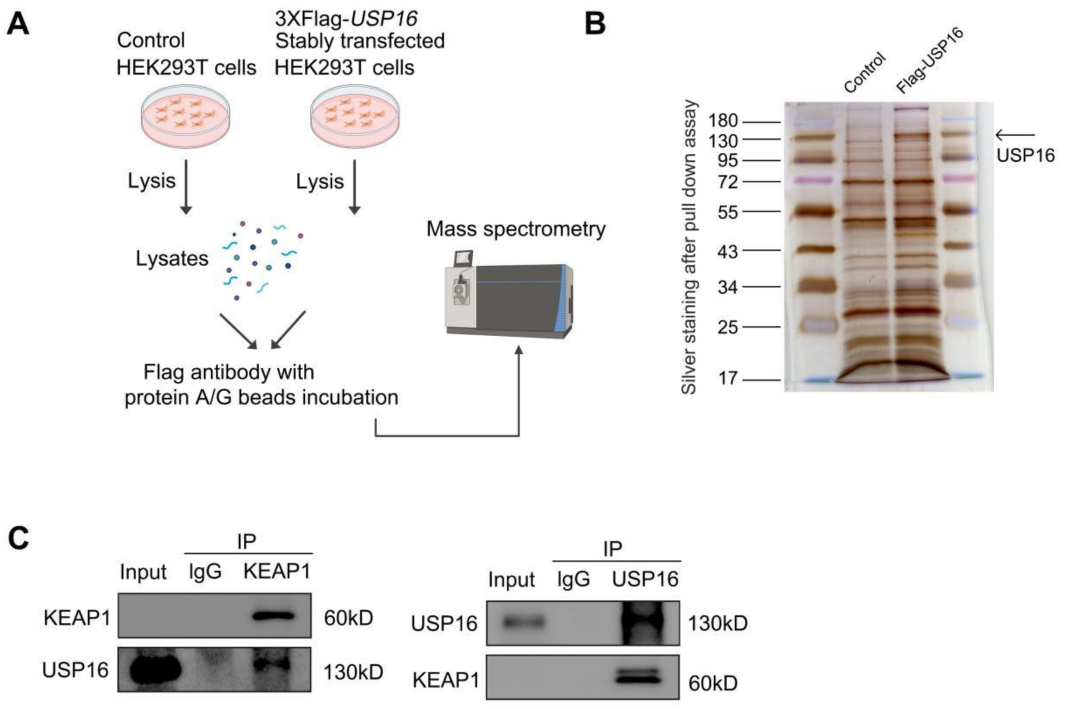

Supplementary Figure 7 (A) HEK293T cells were transfected with Flag-USP16, and cells were performed with co-immunoprecipitation by Flag antibody or IgG with protein A/G beads. Then mass spectrum was used to detect all the proteins connected with Flag-USP16. (Scheme is Created with BioRender.com) (B) Co-immunoprecipitation was carried out in HEK293T cells and then conducting silver staining (n=3). (C) Co-immunoprecipitation assay showed the endogenous interaction between USP16 and KEAP1 in HepG2 cells (n=3). All numbers (n) are biologically independent experiments. Source data are provided as a Source Data file.

Supplementary Figure 8

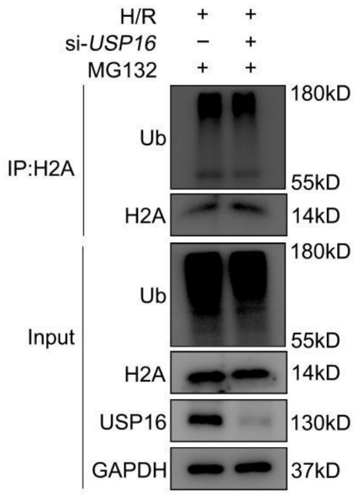

Supplementary Figure 8 (A) The ubiquitination level of H2A was determined in H/R (4 h/6 h) treated HepG2 cells transfected with si-*USP16* or si-*scramble* in the presence of MG132 (10 μM) (n=3). Data are presented as means ± SEM with individual values. All numbers (n) are biologically independent experiments. Source data are provided as a Source Data file.

Supplementary Figure 9

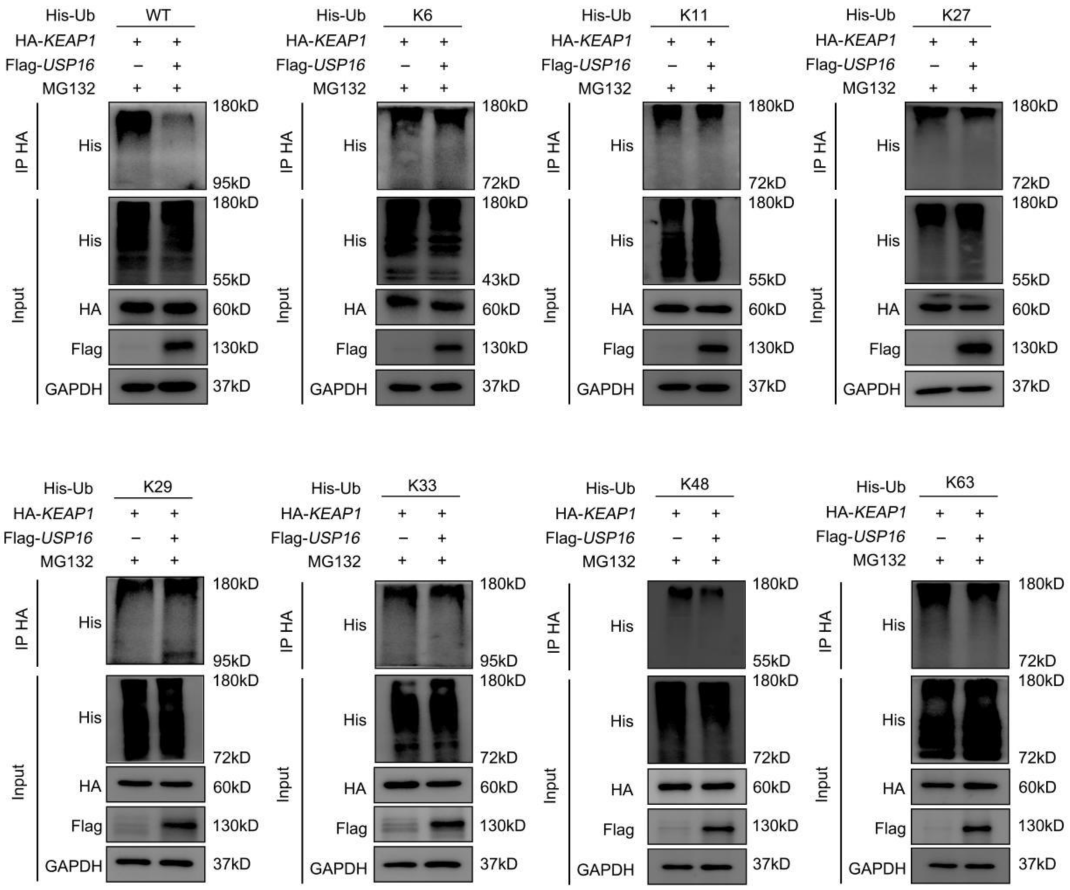

Supplementary Figure 9 (A) HEK293T cells were transfected with HA-KEAP1 and Flag-USP16. Then cells were simultaneously overexpressed with different His-Ub plasmids (His-Ub, K6-Ub, K11-Ub, K27-Ub, K29-Ub, K33-Ub, K48-Ub, and K63-Ub). After 48 h, cells were collected and performed with Co-IP (n=3). Data are presented as means ± SEM with individual values. All numbers (n) are biologically independent experiments. Source data are provided as a Source Data file.

Supplementary Figure 10

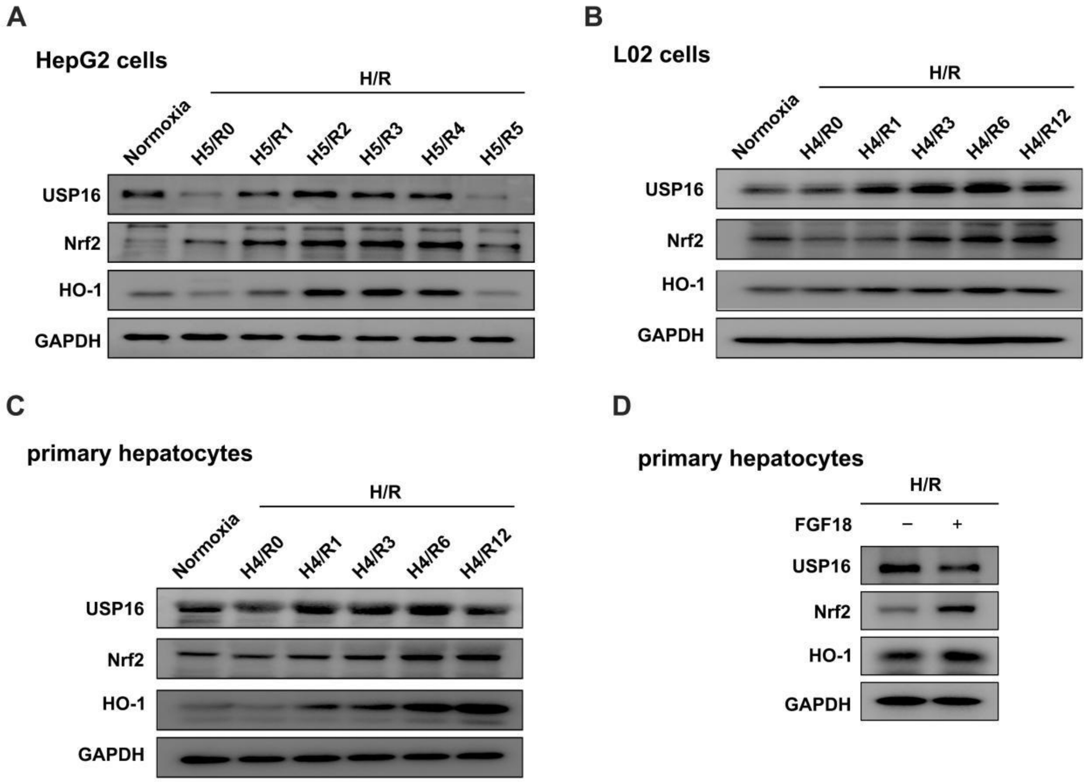

Supplementary Figure 10 The optimal timing of the H/R models in hepatocytes

(A) Protein levels of USP16, Nrf2, and HO-1 in HepG2 cells subjected to different time points of H/R challenge. (B) Protein levels of USP16, Nrf2, and HO-1 in L02 cells subjected to different time points of H/R challenge. (C) Protein levels of USP16, Nrf2, and HO-1 in primary hepatocytes subjected to different time points of H/R challenge (n=3). (D) Protein levels of Nrf2, USP16, and HO-1 in FGF18 (10 ng/mL) treated primary hepatocytes subjected to H/R (4 h/6 h) challenge (n=3). Data are

presented as means ± SEM with individual values. All numbers (n) are biologically independent experiments. Source data are provided as a Source Data file.

Supplementary Figure 11

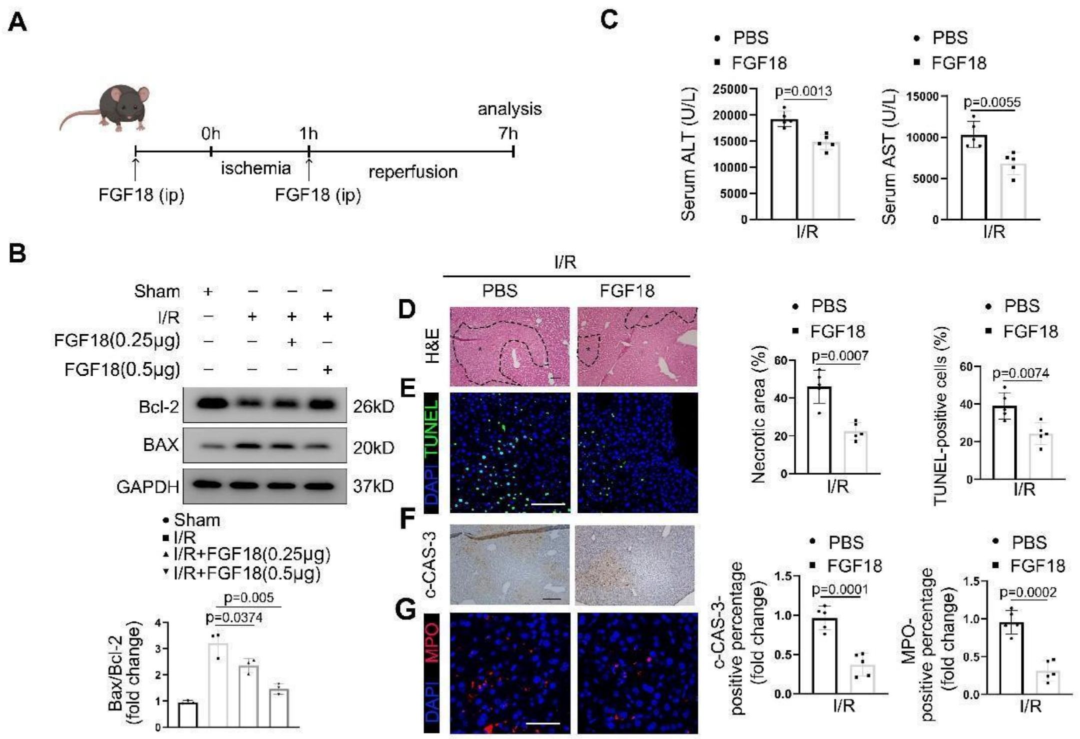

Supplementary Figure 11 FGF18 protein injection alleviates liver damage during hepatic IRI. (A) Schematic diagram of the work. (Scheme is Created with BioRender.com) (B) Relative protein expression by western blotting (n=3). (C) Serum ALT and AST level (n=5). (D) H&E staining of liver sections. Scale bar=100 μm (n=5). (E-G) TUNEL staining, c-CAS-3 immunohistochemistry, and MPO staining of liver sections (n=5). Scale bar=100 μm. The statistical significance of differences were assessed by two-tailed student unpaired t-test for C. Other assays were assessed by one-way ANOVA, followed by Tukey's multiple comparison test. Data are

presented as means ± SEM with individual values. All numbers (n) are biologically independent experiments. Source data are provided as a Source Data file.

.
