# Supplementary material for: FGF18 alleviates hepatic ischemia-reperfusion injury via the USP16-mediated KEAP1/Nrf2 signaling pathway in male mice
Source: Nat Commun. 2023 Sep 30;14:6107. doi: 10.1038/s41467-023-41800-x (PMC10542385; doi:10.1038/s41467-023-41800-x)
Supplement: Supplementary file 3 — Description of Additional Supplementary Files [file 41467_2023_41800_MOESM3_ESM.pdf]

File name: Supplementary Data 1

Description: Summary patient characteristics

File name: Supplementary Data 2

Description: Putative Flag-USP16 interacting proteins

File name: Supplementary Data 3

Description: RNA-seq summary

File name: Supplementary Data 4

Description: Primers for rt-PCR

File name: Supplementary Data 5

Description: Primers for si-RNA sequence

File name: Supplementary Data 6

Description: Antibodies
