# Supplementary material for: FGF18 alleviates hepatic ischemia-reperfusion injury via the USP16-mediated KEAP1/Nrf2 signaling pathway in male mice
Source: Nat Commun. 2023 Sep 30;14:6107. doi: 10.1038/s41467-023-41800-x (PMC10542385; doi:10.1038/s41467-023-41800-x)
Supplement: Supplementary file 10 — Reporting Summary [file 41467_2023_41800_MOESM10_ESM.pdf]

nature portfolio

Corresponding author(s): Weitao Cong

Last updated by author(s): Sep 4, 2023

Reporting Summary

Nature Portfolio wishes to improve the reproducibility of the work that we publish. This form provides structure for consistency and transparency in reporting. For further information on Nature Portfolio policies, see our Editorial Policies and the Editorial Policy Checklist.

Statistics

For all statistical analyses, confirm that the following items are present in the figure legend, table legend, main text, or Methods section.

n/a	Confirmed	
☐	☒	The exact sample size (*n*) for each experimental group/condition, given as a discrete number and unit of measurement
☐	☒	A statement on whether measurements were taken from distinct samples or whether the same sample was measured repeatedly
☐	☒	The statistical test(s) used AND whether they are one- or two-sided<br>*Only common tests should be described solely by name; describe more complex techniques in the Methods section.*
☒	☐	A description of all covariates tested
☒	☐	A description of any assumptions or corrections, such as tests of normality and adjustment for multiple comparisons
☐	☒	A full description of the statistical parameters including central tendency (e.g. means) or other basic estimates (e.g. regression coefficient) AND variation (e.g. standard deviation) or associated estimates of uncertainty (e.g. confidence intervals)
☐	☒	For null hypothesis testing, the test statistic (e.g. *F*, *t*, *r*) with confidence intervals, effect sizes, degrees of freedom and *P* value noted<br>*Give P values as exact values whenever suitable.*
☒	☐	For Bayesian analysis, information on the choice of priors and Markov chain Monte Carlo settings
☒	☐	For hierarchical and complex designs, identification of the appropriate level for tests and full reporting of outcomes
☒	☐	Estimates of effect sizes (e.g. Cohen's *d*, Pearson's *r*), indicating how they were calculated

Our web collection on statistics for biologists contains articles on many of the points above.

Software and code

Policy information about availability of computer code

Data collection	1. LC-MS /MS experiments were performed on a Q Exactive HF-X mass spectrometer that was coupled to Easy nLC1200(Thermo Scientific).<br>2. The protein bands data was collected by exposure machine (GE, Amersham154 imager 680).<br>3. The fluorescence image data was collected by confocal microscope (Leica TCS SP8).<br>[illegible]
Data analysis	The protein bands data, pcr data, and fluorescence data were analyzed by imageJ software 1.51j8.<br>The mass data were analyzed using MaxQuant software version 1.6.1.0. MS data were searched against the UniPorKB Rattus norvegicus database (36080 total entries)

For manuscripts utilizing custom algorithms or software that are central to the research but not yet described in published literature, software must be made available to editors and reviewers. We strongly encourage code deposition in a community repository (e.g. GitHub). See the Nature Portfolio guidelines for submitting code & software for further information.

Data

Policy information about availability of data

All manuscripts must include a data availability statement. This statement should provide the following information, where applicable:

- Accession codes, unique identifiers, or web links for publicly available datasets
- A description of any restrictions on data availability
- For clinical datasets or third party data, please ensure that the statement adheres to our policy

The public RNA-seq used in this study are available in the GSE93034 (USP) AND SRP117594 (FGF). The mass spectrometry proteomics data have been deposited to the Proteosited to the ProteomeXchange Consortium via the PRIDE partner repository with the dataset identifier PXD045021.The RNA-seq data is deposited in GEO with the accession code of GSE242032.
Source data containing uncropped blots and raw data containing uncropped blots and raw data for all plots are provided with this paper. All other data supporting the findings of this study are available within the article and its supplementary files.Source data are provided with this paper.

Research involving human participants, their data, or biological material

Policy information about studies with human participants or human data. See also policy information about sex, gender (identity/presentation), and sexual orientation and race, ethnicity and racism.

Reporting on sex and gender	The ratio of men to wowen in this experiment is 24/8. And informed consent forms were signed by all patients. We did not explore the effect of sex on the experiment.
Reporting on race, ethnicity, or other socially relevant groupings	N/A
Population characteristics	All the information about antidodies were provided in Supplementary Supplementary Data 1.
Recruitment	The tissues used in this experiment were samples from partial liver resections.Perhaps samples from liver transplants will also need to be explored to be explored in future studies.
Ethics oversight	All liver samples were obtained under protocols approved by 2nd Affiliated Hospital of wenzhou medical university(Institutional Review Board approval number 2021-K-106-01).

Note that full information on the approval of the study protocol must also be provided in the manuscript.

Field-specific reporting

Please select the one below that is the best fit for your research. If you are not sure, read the appropriate sections before making your selection.

☒ Life sciences ☐ Behavioural & social sciences ☐ Ecological, evolutionary & environmental sciences

For a reference copy of the document with all sections, see nature.com/documents/nr-reporting-summary-flat.pdf

Life sciences study design

All studies must disclose on these points even when the disclosure is negative.

Sample size	We chose the sample sizes based on the previous studies in the model of hepatic ischemia reperfusion. PMID:32343849, PMID:33445067.
Data exclusions	No data were excluded from the analyses.
Replication	Experiments in the article are reliably produced ,replication were described in the figure legends.
Randomization	All mice were randomly assigned to group, using a randomization procedure (http:www.randomizer.org/))
Blinding	Yes, the investigators were blinded to group allocation during data collection and analysis.

Behavioural & social sciences study design

All studies must disclose on these points even when the disclosure is negative.

Study description	
Research sample	

Sampling strategy

Data collection

Timing

Data exclusions

Non-participation

Randomization

Ecological, evolutionary & environmental sciences study design

All studies must disclose on these points even when the disclosure is negative.

Study description

Research sample

Sampling strategy

Data collection

Timing and spatial scale

Data exclusions

Reproducibility

Randomization

Blinding

Did the study involve field work? ☐ Yes ☐ No

Field work, collection and transport

Field conditions

Location

Access & import/export

Disturbance

Reporting for specific materials, systems and methods

We require information from authors about some types of materials, experimental systems and methods used in many studies. Here, indicate whether each material, system or method listed is relevant to your study. If you are not sure if a list item applies to your research, read the appropriate section before selecting a response.

Materials & experimental systems

n/a	Involved in the study
☐	☒ Antibodies
☐	☒ Eukaryotic cell lines
☒	☐ Palaeontology and archaeology
☐	☒ Animals and other organisms
☒	☐ Clinical data
☒	☐ Dual use research of concern
☒	☐ Plants

Methods

n/a	Involved in the study
☒	☐ ChIP-seq
☒	☐ Flow cytometry
☒	☐ MRI-based neuroimaging

Antibodies

Antibodies used

Antibodies were obtained from cell signaling technology: FGFR1(9740,1:1000),FGFR2(23328,1:1000),FGFR3(4574,1:1000),FGFR4(8562,1:1000),BAX(2772,1;1000),c-CAS-3(9661,1:1000),aSMA(19245,1:200),LYVE-1(6753,1:200),F4/80(30325,1:400);
Antibodies were obtained from Huabio: Bcl-2(ET1702-53,1:1000),Desmin(ET1606-30,1:200),Albumin(0806-9, 1:200), GFAP(ET140707,!:200);
Antibodies were obtained from Proteintech: Nrf2(16396-1-AP,1:1000),ho-1(10701-1-AP,1:1000),KEAP1(10503-2-AP,1:1000),USP16(14055-1-AP,1:1000),HA(51064-2-AP,1:1000),Flag(66008-4-ig,1:1000).
Antibodies were obtained from santa cruz: FGF18(393491,1:1000),KEAP1(514914,1:100),USP16(390683,1:200),His(8036,1:1000),ubiquitin(8017,1:1000),Flag(166355,1:100),HA (7392,1:100), IgG(B0619,1:100).
Antibodies were obtianed from abcam: nrf2(31163,1:1000),MPO(208670,1:200),4-HNE(48506,1:200),vimentin(196579,1:200).
Antibodies were obtained from abclonal: H2A(a3692,1:1000), CD68(196579,1:200).

Validation

Antibodies critical for novel conclusions were validated by elimination of signals upon knock-down experiments and/or functional assays. All antibodies were used in the system under study (assay and species) according to the profile of manufacturer.

Eukaryotic cell lines

Policy information about cell lines and Sex and Gender in Research

Cell line source(s)

In this studies, we use L02 cells?HepG2 cells?HEK293T cells?LX-2 cells?and primary liver cells(male mice). L02 cells, HepG2 cell, HEK293T cell, and LX-2 cell were purchased from procell(Wuhan).

Authentication

All cell lines are commericial and no authentication has been conducted after purchase.

Mycoplasma contamination

All cell lines were tested to be mycoplasma contamination negative.

Commonly misidentified lines
(See ICLAC register)

No cell lines usded in this study were found in the database of commonly misdentified cell lines that in maintained by ICLAC.

Palaeontology and Archaeology

Specimen provenance

Specimen deposition

Dating methods

☐ Tick this box to confirm that the raw and calibrated dates are available in the paper or in Supplementary Information.

Ethics oversight

Note that full information on the approval of the study protocol must also be provided in the manuscript.

Animals and other research organisms

Policy information about studies involving animals; ARRIVE guidelines recommended for reporting animal research, and Sex and Gender in Research

Laboratory animals

Age 6-8 weeks Usp16f/f ?Fgf18f/f?GFAP-Cre?Nrf2 KO and fgf18-tdtomato. All the transgenic mice were on a C57BL/6 background.All mice were housed in constant temperature(22±2) and humidity(40-60%) with free access to food and water.

Wild animals

the study did not involve wild animals.

Reporting on sex	the study only use male mice.
Field-collected samples	No fieid-collected samples were used in this study.
Ethics oversight	All experimental procedures and methods were approved by the institutional Animal Care and Use Committee of Wenzhou Medical University.

Note that full information on the approval of the study protocol must also be provided in the manuscript.

Clinical data

Policy information about clinical studies

All manuscripts should comply with the ICMJE guidelines for publication of clinical research and a completed CONSORT checklist must be included with all submissions.

Clinical trial registration	
Study protocol	
Data collection	
Outcomes	

Dual use research of concern

Policy information about dual use research of concern

Hazards

Could the accidental, deliberate or reckless misuse of agents or technologies generated in the work, or the application of information presented in the manuscript, pose a threat to:

No	Yes	
☐	☐	Public health
☐	☐	National security
☐	☐	Crops and/or livestock
☐	☐	Ecosystems
☐	☐	Any other significant area

Experiments of concern

Does the work involve any of these experiments of concern:

No	Yes	
☐	☐	Demonstrate how to render a vaccine ineffective
☐	☐	Confer resistance to therapeutically useful antibiotics or antiviral agents
☐	☐	Enhance the virulence of a pathogen or render a nonpathogen virulent
☐	☐	Increase transmissibility of a pathogen
☐	☐	Alter the host range of a pathogen
☐	☐	Enable evasion of diagnostic/detection modalities
☐	☐	Enable the weaponization of a biological agent or toxin
☐	☐	Any other potentially harmful combination of experiments and agents

Plants

Seed stocks	
Novel plant genotypes	
Authentication	

ChIP-seq

Data deposition

- [ ] Confirm that both raw and final processed data have been deposited in a public database such as GEO.
- [ ] Confirm that you have deposited or provided access to graph files (e.g. BED files) for the called peaks.

Data access links<br>*May remain private before publication.*	
Files in database submission	
Genome browser session<br>(e.g. UCSC)	

Methodology

Replicates	
Sequencing depth	
Antibodies	
Peak calling parameters	
Data quality	
Software	

Flow Cytometry

Plots

Confirm that:

- [ ] The axis labels state the marker and fluorochrome used (e.g. CD4-FITC).
- [ ] The axis scales are clearly visible. Include numbers along axes only for bottom left plot of group (a 'group' is an analysis of identical markers).
- [ ] All plots are contour plots with outliers or pseudocolor plots.
- [ ] A numerical value for number of cells or percentage (with statistics) is provided.

Methodology

Sample preparation	
Instrument	
Software	
Cell population abundance	
Gating strategy	

- [ ] Tick this box to confirm that a figure exemplifying the gating strategy is provided in the Supplementary Information.

Magnetic resonance imaging

Experimental design

Design type	
Design specifications	
Behavioral performance measures	

Acquisition

Imaging type(s)

Field strength

Sequence & imaging parameters

Area of acquisition

Diffusion MRI ☐ Used ☐ Not used

Preprocessing

Preprocessing software

Normalization

Normalization template

Noise and artifact removal

Volume censoring

Statistical modeling & inference

Model type and settings

Effect(s) tested

Specify type of analysis: ☐ Whole brain ☐ ROI-based ☐ Both

Statistic type for inference

(See Eklund et al. 2016)

Correction

Models & analysis

n/a	Involved in the study
☐	☐ Functional and/or effective connectivity
☐	☐ Graph analysis
☐	☐ Multivariate modeling or predictive analysis

Functional and/or effective connectivity

Graph analysis

Multivariate modeling and predictive analysis
